# Comparative transcriptome analysis of second- and third-generation merozoites of *Eimeria necatrix*

**DOI:** 10.1186/s13071-017-2325-z

**Published:** 2017-08-16

**Authors:** Shijie Su, Zhaofeng Hou, Dandan Liu, Chuanli Jia, Lele Wang, Jinjun Xu, Jianping Tao

**Affiliations:** 1 College of Veterinary Medicine, Yangzhou University, Yangzhou, 225009 China; 2 Jiangsu Co-innovation Center for Prevention and Control of Important Animal Infectious Diseases and Zoonoses, Yangzhou University, Yangzhou, 225009 China; 3 Jiangsu Key Laboratory of Zoonosis, Yangzhou University, Yangzhou, 225009 China

**Keywords:** *Eimeria*, Merozoite, RNA-seq, Comparative transriptomes, Differentially expressed genes

## Abstract

**Background:**

*Eimeria* is a common genus of apicomplexan parasites that infect diverse vertebrates, most notably poultry, causing serious disease and economic losses. *Eimeria* species have complex life-cycles consisting of three developmental stages. However, the molecular basis of the *Eimeria* reproductive mode switch remains an enigma.

**Methods:**

Total RNA extracted from second- (MZ-2) and third-generation merozoites (MZ-3) of *Eimeria necatrix* was subjected to transcriptome analysis using RNA sequencing (RNA-seq) followed by qRT-PCR validation.

**Results:**

A total of 6977 and 6901 unigenes were obtained from MZ-2 and MZ-3, respectively. Approximately 2053 genes were differentially expressed genes (DEGs) between MZ-2 and MZ-3. Compared with MZ-2, 837 genes were upregulated and 1216 genes were downregulated in MZ-3. Approximately 95 genes in MZ-2 and 48 genes in MZ-3 were further identified to have stage-specific expression. Gene ontology category and KEGG analysis suggested that 216 upregulated genes in MZ-2 were annotated by 70 GO assignments, 242 upregulated genes were associated with 188 signal pathways, while 321 upregulated genes in MZ-3 were annotated by 56 GO assignments, 322 upregulated genes were associated with 168 signal pathways. The molecular functions of upregulated genes in MZ-2 were mainly enriched for protein degradation and amino acid metabolism. The molecular functions of upregulated genes in MZ-3 were mainly enriched for transcriptional activity, cell proliferation and cell differentiation.

**Conclusions:**

To the best of our knowledge, this is the first RNA-seq data study of the MZ-2 and MZ-3 stages of *E*. *necatrix*; it demonstrates a high number of differentially expressed genes between the MZ-2 and MZ-3 of *E*. *necatrix*. This study forms a basis for deciphering the molecular mechanisms underlying the shift from the second to third generation schizogony in *Eimeria*. It also provides valuable resources for future studies on *Eimeria*, and provides insight into the understanding of reproductive mode plasticity in different *Eimeria* species.

**Electronic supplementary material:**

The online version of this article (doi:10.1186/s13071-017-2325-z) contains supplementary material, which is available to authorized users.

## Background

Avian coccidiosis is a major worldwide veterinary health challenge caused by the obligate intracellular protozoan parasites of the phylum Apicomplexa, *Eimeria*. Clinical coccidiosis causes a reduction in weight gain, ineffective feed-conversion, and high levels of mortality. Although coccidiosis is a well-known disease, it still remains one of the most economically important parasitic diseases of the poultry industry throughout the world. It has been estimated that the total annual cost for avian coccidiosis control is approximately $30–60 million in China [1], and the global cost is likely greater than $800 million annually [2]. With the development of drug resistance in *Eimeria* species threatening the continued use of prophylactic anticoccidials, vaccination remains a desirable long-term strategy for combatting this disease [3].

The life-cycle of *Eimeria* spp. is the most straight-forward of any apicomplexan parasite and includes merogony, gametogony and sporogony. Parasites are transmitted *via* sporulated oocysts. Once ingested, sporozoites are released from the oocysts into intestinal epithelial cells. Several asexual cycles result in the growth of merozoites, which differentiate into the sexual stages. Female macrogametes are fertilized by male microgametes to produce oocysts, which are shed in the feces of infected hosts. After sporulation in the external environment, oocysts can infect other animals once consumed. At different developmental stages, the genes expressed, as well as the levels of gene expression, vary. Although the second- and third-generation meronts and merozoites belong to the asexual reproduction stage, the mature second- and third-generation *E. tenella* merozoites and meronts can be differentiated by periodic acid Schiff’s (PAS) staining, concluded by Klimes et al. [4]. Cornelissen et al. [5, 6] also found that the two generation merozoites of *E. tenella* could be differentiated by Feulgen-pararosaniline staining. These reports indicated that the second- and third-generation merozoites had already been sexually differentiated. The differentiation and development of different biological stages are dependent on the regulation of gene transcription. A few stage-specific genes have been identified in oocysts, sporozoites and second-generation merozoites of *E. tenella*, *E. maxima* and *E*. *acervulina* [7–9] in tachyzoites, sporozoites and oocysts of *Toxoplasma gondii* [10–13] and in human and mosquito stages [14] and gametocyte stages of *Plasmodium falciparum* [15], based on profiling quantitative changes in gene transcription. To date, a global analysis of gene transcription has not yet to be performed for every stage of the coccidian life-cycle, due in part to difficulties in producing sufficient quantities of parasitic material for conventional transcriptional analysis.

*Eimeria necatrix* is a highly pathogenic coccidium that can cause high mortality in susceptible birds. Coccidiosis caused by *E*. *necatrix* mainly occurs in chickens older than 8 weeks when raised on a litter floor [16, 17]. Compared with other species of avian coccidia, the life-cycle of *E*. *necatrix* is somewhat distinct. Its first- and second-generation meronts are primarily located in the mid-intestinal area of host chickens, and its third-generation meronts, and later oocyst development, occur only in the cecum [18]. Therefore, it is relatively easy to obtain sufficient quantities of the second- and third-generation merozoites through isolation from the mid-intestine and cecum, respectively, of chickens.

To the best of our knowledge, there are no reports to date concerning analysis of the transcriptome of the second- and third-generation merozoites of *E*. *necatrix*, nor of its stage-specific genes. The process of MZ-2 conversion to MZ-3 is confusing, and an issue on whether sex development and differentiation has happened in MZ-3 is also not clear. In the present study, therefore, we conducted RNA sequencing using a next-generation sequencer to identify differentially expressed gene (DEG) profiling from the second- and third-generation merozoites of *E*. *necatrix*. To gain an understanding of the biology of the differentiation and development of *E*. *necatrix*, we determined the expressed mRNAs at these stages and the relevant metabolic pathways based on information from other species in the GenBank database. The putative roles of selected DEGs and significantly enriched signal pathways are potentially involved in the switching from the second to the third generation schizogony and the sex differentiation and development. This study provides the most comprehensive dataset to date for gene expression in different development stages of *E*. *necatrix*, which could facilitate our understanding of molecular mechanisms in reproductive mode switch of *Eimeria*.

## Methods

### Parasite materials

One-day-old chicks (Suqiu Yellow chickens; the Poultry Institute of China Agricultural Academy, Yangzhou, Jiangsu, China) were reared in a coccidia-free isolation facility and allowed unlimited access to water and food that did not contain any anti-coccidial drugs or antibiotics. To confirm chickens as free of infection prior to experimental inoculation, feces were analyzed by salt-flotation and light microscopy to ensure the absence of oocysts [19]. Chickens at 4 weeks of age were orally infected with 2.0 × 10^4^ sporulated oocysts of *E*. *necatrix*. The Yangzhou strain of *E*. *necatrix*, originally isolated from chickens that died from *E*. *necatrix* infection by the Key Laboratory for Avian Preventive Medicine at Yangzhou University, was used in this study. All animal care and procedures were conducted according to the guidelines for animal use in toxicology. The study protocol was approved by the Animal Care and Use Committee of the College of Veterinary Medicine, Yangzhou University.

### Preparation of merozoites

#### Second-generation merozoites (MZ-2)

At 136 h post-infection (HPI), chickens were sacrificed, and their intestines were removed and cut open to clean out the intestinal contents by rinsing in cold PBS. The mucosa was scraped using a glass slide. All the collected scrapings were ground down and incubated for 60 min at 37 °C in digestion liquid (120 mM NaCl, 10 mM CaCl_2_, 3 mM K_2_HPO_4_, 20 mM Tris-HCl, 0.1% BSA, 0.1% hyaluronidase). Large intestinal debris was then removed by filtering through gauze, and smaller debris removed by sequential filtration through 17 μm and 10 μm polymon mesh (Sefar Filtration Solution Co. Ltd., Suzhou, China). The merozoites were centrifuged at 3000 rpm for 10 min, the supernatant was discarded, and the merozoite pellet was re-suspended in PBS. After the centrifugation and washing steps were repeated two times, the merozoite pellet was re-suspended in red blood cell lysis buffer (Solarbio, Beijing, China) and allowed to sit at 4 °C for 10 min. The merozoites were then washed with cold PBS three times. The resulting merozoites were purified by density-gradient centrifugation using the method described by Mo et al. [20]. Approximately l0^10^ merozoites were recovered from each chicken (Additional file 1: Figure S1a). Purified merozoites were stored at -80 °C for further use.

#### Third-generation merozoites (MZ-3)

At 144 HPI, chickens were sacrificed, and their ceca were removed and cut open for cleaning of the cecal contents by rinsing in cold PBS. The mucosa in the distal region of the ceca, approximately 3 cm from the ileo-cecal junction, was scraped using a glass slide. All the collected scrapings were processed for merozoite isolation as described above. The resulting merozoites were passed through a DEAE-52 cellulose chromatographic column as described by Chapman et al. [21]. Approximately l0^7^ merozoites were recovered from each chicken (Additional file 1: Figure S1b). Purified merozoites were stored at -80 °C for further use.

### RNA extraction and purification

Total RNA was extracted using RNAiso Plus Total RNA extraction reagent (Takara Dalian,China), according to the manufacturer’s instructions. The quality of the RNA was measured using a NanoDrop ND-2000, and the integrity was evaluated with an Agilent Bioanalyzer 2100 (Agilent Technologies, Santa Clara, CA, USA). Only samples with RNA Integrity Numbers (RIN) from 7 to 10 were used for further study. Qualified total RNA was further purified with an RNeasy micro kit (Qiagen, Hilden, Germany) and an RNase-Free DNase Set (Qiagen, Hilden, Germany) and then re-assessed on a Bioanalyzer 2100 (Agilent Technologies, Santa Clara, CA, USA).

### cDNA library construction and RNA-seq

Illumina sequencing libraries were constructed using a TruSeq™ RNA Sample Pre Kit (Illumina, San Diego, CA, USA). In brief, 1–3 μg of purified total RNA was used for isolation of coding RNA and non-coding RNA using a ribo-zero kit for rRNA depletion, and isolated RNA samples were simultaneously eluted and fragmented in Elute, Prime, Fragment Mix at 94 °C for 8 min to obtain 120–200 bp inserts. First-strand cDNA was synthesized with SuperScript II Reverse Transcriptase (Invitrogen, Carlsbad, CA, USA) in the presence of random hexamer primers. Second-strand cDNA synthesis was carried out at 16 °C for 1 h. After polyadenylation of the 3′ end, the DNA fragments were ligated with TruSeq adapters, and Agencourt ® Ampure XP beads (Beckman, Brea, USA) were used to isolate the double-strand (ds) cDNA synthesized by the Second Strand Master Mix. The samples were amplified with TruSeq PCR primers. DNA size and purity of the cDNA library were checked using a high-sensitivity DNA 1000 kit on a Bioanalyzer 2100 system (Agilent Technologies, Santa Clara, CA, USA). The quantification of the cDNA libraries was performed with a Qubit™ dsDNA HS Assay kit on a Qubit® 2.0 Fluorometer (Life Technologies, Carlsbad, CA, USA). Cluster generation was performed on a paired-end flow cell using a cBot Clonal Amplification System with a HiSeq PE Cluster Kit v4 (Illumina, San Diego, CA, USA), and sequenced on the Illumina HiSeq 2500 platform using the SBS 36-cycle Sequencing Kit (v5) at Shanghai Biotechnology Corporation (Shanghai, China) according to manufacturer-recommended cycling parameters. According to the manufacturer’s instructions, paired reads were sequenced using the double-read multiplex program on the Illumina HiSeq 2500 platform at Shanghai Biotechnology Corporation. Each library was run on two lanes in a flow cell in order to maximize the total number of RNA-seq reads.

### Sequence data analysis

Raw reads in fastq format were filtered, and the adaptor sequences and low quality reads were removed. Stringent filtering criteria were used to minimize the effects of sequencing errors during the assembly. Briefly, bases with a 3 terminal quality score lower than 10 and a read-length shorter than 20 bp were discarded. Genome mapping was performed based on the recently published *E*. *necatrix* Houghton strain reference genome sequence (GCA_000499385.1) using Tophat (version 2.0.9) with a spliced-mapping algorithm. After genome mapping, reads with less than two-base mismatches and multi hits ≤ 2 were retained.

### Identification of differentially expressed genes (DEGs)

The mapped fragments were normalized for RNA length according to the fragment per kilobase of exon model per million mapped reads (FPKM) for each gene [22], which facilitated the comparison of transcript levels between samples. Differentially expressed genes (DEGs) between the two samples were selected using the following filter criteria: FDR (False discovery rate) ≤ 0.05 and the Fold-change ≥ 2 or log_2_ (FPKM ratio of two samples) ≥ 1. Furthermore, the enrichment analysis of DEGs was conducted with the GO database (http://www.geneontology.org/), and the gene number for each GO term was calculated. The main pathways of biochemical and signal transduction significantly associated with DEGs were determined *via* KEGG pathway analysis.

### Relative quantitative real-time PCR (qRT-PCR) analysis

To confirm the transcription levels of genes identified by RNA-seq, 9 genes (3 upregulated genes, 3 downregulated genes and 3 with no significant difference), based on their involvement in different expression patterns between MZ-2 and MZ-3, were measured using qRT-PCR. The mRNA was treated with a PrimeScript™ RT reagent Kit with gDNA Eraser (Perfect Real Time) (Takara, Dalian, China) to digest genomic DNA and synthesize cDNA according to the manufacturer’s instructions. The primers used for qRT-PCR were designed according to the RNA-seq data with Primer Premier 5.0 software (Premier Biosoft International, Palo Alto, CA, USA). Glyceraldehyde-3-phosphate dehydrogenase (GAPDH) was used as an endogenous marker to normalize the reactions to the same amplification progression. By employing FastStart Universal SYBR Green Master (Roche, Shanghai, China), qRT-PCR was performed following the manufacturer’s protocols. Each 20 μl qRT-PCR reaction mixture comprised a 2 μl dilution of cDNA, 10 μl 2 × SYBR Green Master and 4 μl (10 μM) of each primer. PCR amplification was performed with the following cycling parameters: 95 °C for 5 min, followed by 45 cycles of 94 °C for 10 s, 60 °C for 10 s, and 72 °C for 15 s. Melting curve analysis was performed at the end of the PCR amplification over the range of 60–95 °C, increasing the temperature stepwise by 0.5 °C every 10 s. After amplification, the relative fold change of the differentially expressed genes was calculated through the 2^-ΔΔCt^ method [23]. All quantitative PCR analyses for each gene used three biological replicates, with three technical replicates per experiment to guarantee the reproducibility of the amplification. The RNA samples for qRT-PCR were the same as those for Illumina sequencing. Differences were considered statistically significant at *P* < 0.05. All primers are listed in Additional file 2: Table S1.

## Results

### Illumina sequencing and assembly of sequence reads

Three individuals’ RNA samples that show good RNA integrity of the same group were equally mixed to generate an RNA pool, then treated with DNase and evaluated for quality by automated gel electrophoresis. Paired-end sequencing was carried out on all RNA samples using the Illumina HiSeq platform. Approximately 68,009,332 and 51,854,332 raw reads were obtained from MZ-2 and MZ-3 cDNA library, respectively. After removal of low-quality regions, adaptors and all possible contamination, 64,671,002 and 49,249,340 high-quality clean reads were obtained from MZ-2 and MZ-3 samples, respectively. The majority of the clean reads were distributed in the exon region, followed by the intergenic region and the intron region. Clean reads were mapped to the *E*. *necatrix* genome scaffold (https://www.ncbi.nlm.nih.gov/nuccore/NW_013651811.1). A total of 1,537,041 and 979,262 multi reads obtained for MZ-2 and MZ-3, respectively, were mapped to the *E*. *necatrix* genome scaffold. The mapping ratio of two different samples was higher than 66%. After subsequent assembly, a total of 6977 and 6901 unigenes were obtained for MZ-2 and MZ-3, respectively (Table 1).

**Table 1:** Summary of results of MZ-2 and MZ-3 transcriptome

Samples	Raw reads	Clean reads	Mapped reads	Uniquely mapped reads	Multi-mapped reads	Uingenes
MZ-2	68,009,332	64,671,002	43,112,897	41,575,856	1,537,041	6977
MZ-3	51,854,332	49,249,340	33,302,555	32,323,293	979,262	6901

### Annotation

#### BLAST

Gene annotation was based on sequence similarity searches in the National Center for Biotechnology Information database (NCBI) using the BLAST algorithm. A total of 7415 unigenes showed similarities to existing GenBank entries. The results included gene ID, alignment GenBank ID, description in NCBI database, gene short name, locus, FPKM and count. Among the 7415 unigenes, 4749 genes coding for hypothetical proteins were identified.

Normalized read counts were subsequently generated for each of the two different biological stages using the DESeq algorithm [24], thereby allowing a comparison of gene transcript levels between MZ-2 and MZ-3. To analyze the expressional difference between the MZ-2 and MZ-3 stages, the expression level of each unigene in MZ-2 was compared with that in MZ-3.

**Fig. 1:**
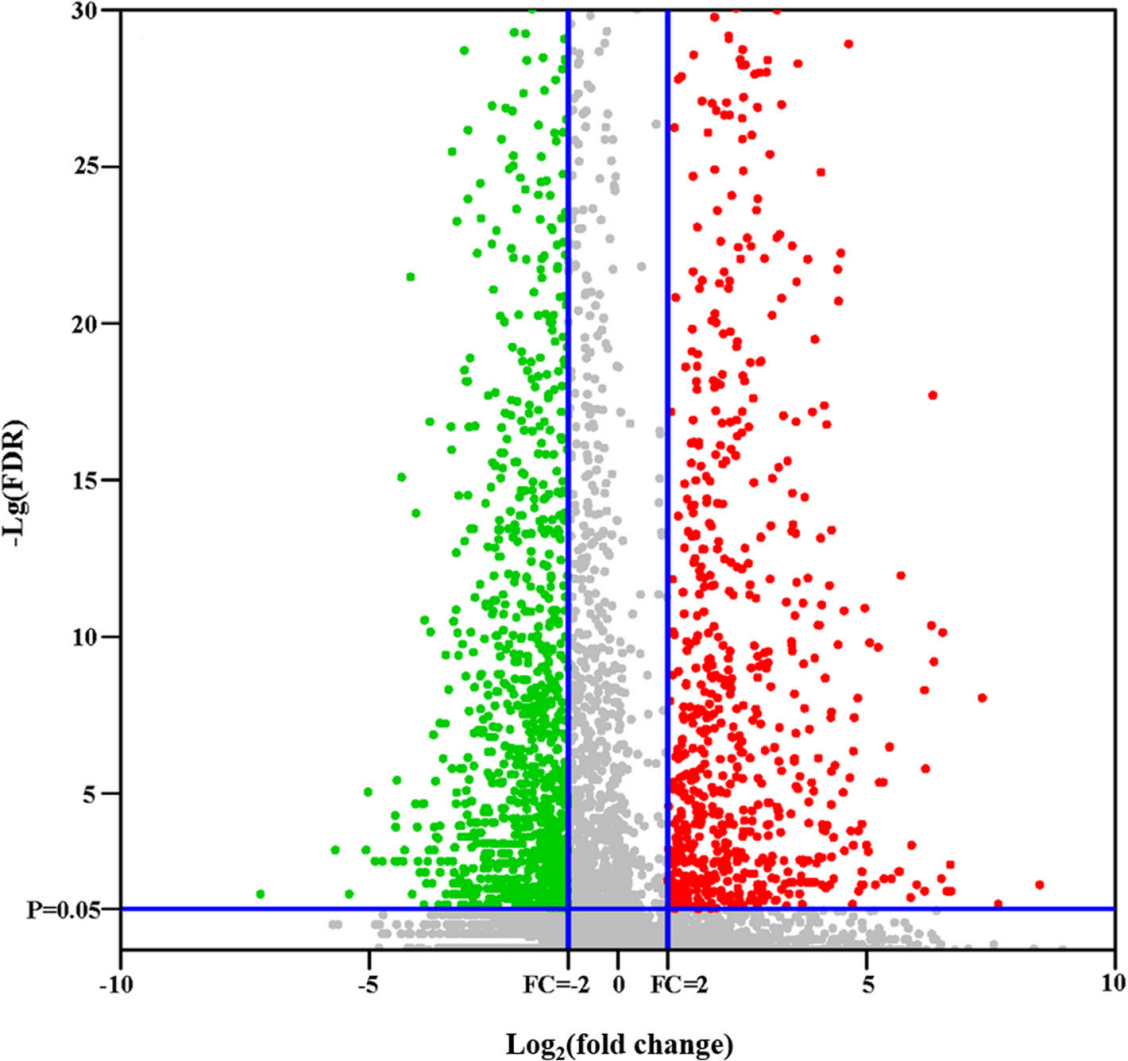
Volcano plots of up/downregulated and not differently expressed unigenes between MZ-2 and MZ-3. Unigenes differently expressed level: the unigenes are significantly upregulated (*red spot*), downregulated (*green spot*), and no difference (*grey spot*)

**Fig. 2:**
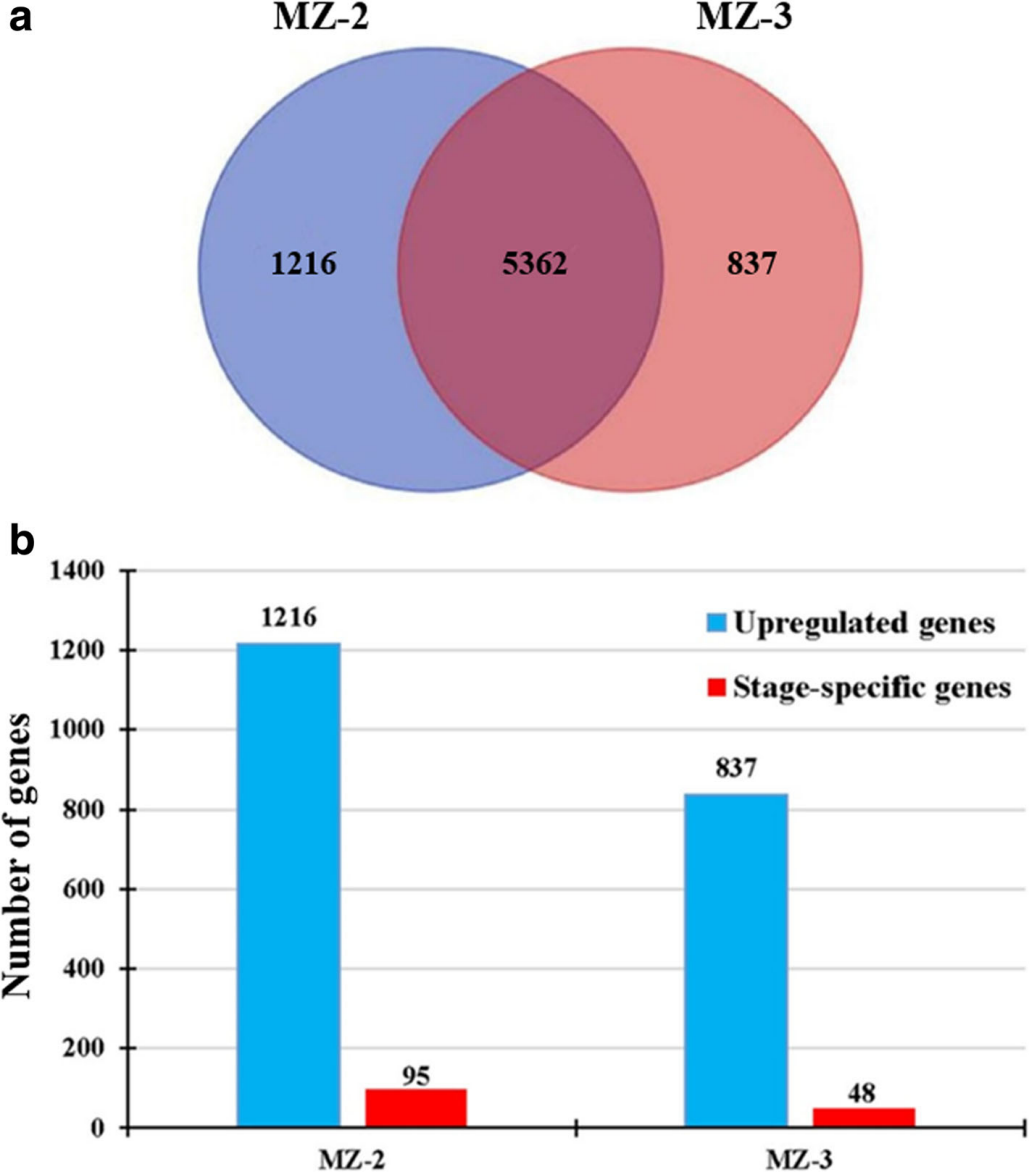
Identification and analysis of upregulated genes in MZ-2 and MZ-3. **a** Venn diagram revealed the overlap between the genes that were upregulation in MZ-3 (*right*) and upregulation in MZ-2 (*left*). A total of 5362 genes were identified in this overlapping region. **b** Bar graph revealed the genes that were upregulation (*blue bar*) and stage-specific expression (*red bar*) in MZ-2 or MZ-3

As shown in volcano plots of up/downregulated genes (Fig. 1), genes were identified as significantly upregulated genes with a log_2_ fold-change ≥ 1 or a fold-change ≥ 2 (red spot), and significantly downregulated genes were defined by a log_2_ fold-change ≤ -1 or a fold-change ≤ -2 (green spot). The comparisons showed that a large population of genes was detected specifically in either MZ-2 or MZ-3. Considering only genes with *P* - values < 2.2e-16, a total of 2053 genes (27.69%) were found to be DEGs between the MZ-2 and MZ-3 stages (Additional file 3: Table S2). Excluding genes with DE false discovery rates of > 0.05, 837 significantly upregulated genes (11.29%) and 1216 significantly downregulated genes (16.40%) were visualized in the Venn diagrams (Fig. 2a). To amplify the difference between the MZ-2 and MZ-3, we defined those DEGs with an FPKM > 1 in MZ-2 or MZ-3 and a FPKM < 1 in MZ-3 or MZ-2 as stage-specific genes. As shown in Fig. 2b, 95 genes (7.81%) were observed to be specifically expressed in MZ-2 (Additional file 4: Table S3), and 48 genes (5.73%) had stage-specific expression in MZ-3 (Additional file 5: Table S4).

### Gene ontology classification

**Fig. 3:**
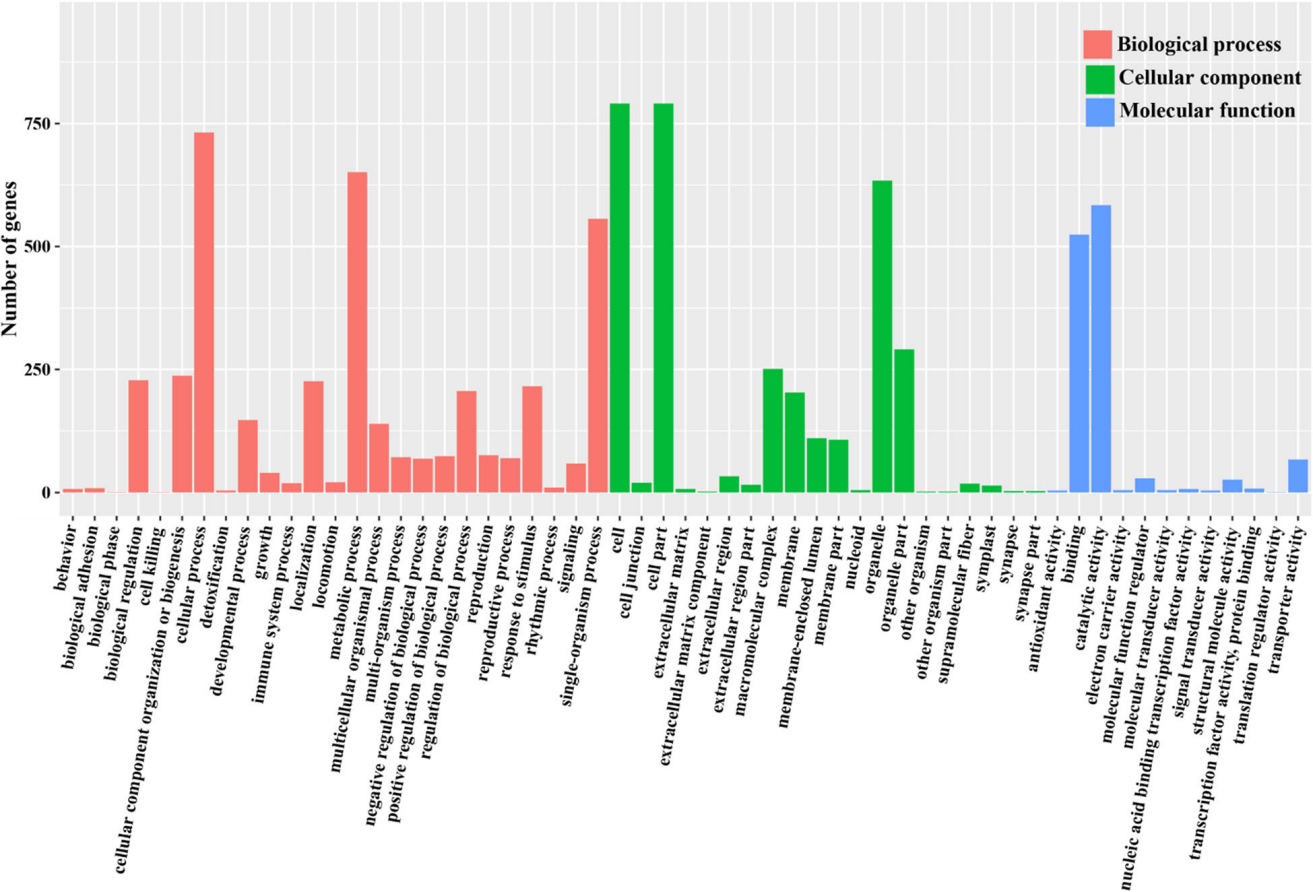
GO analysis of differently expressed genes. The results are divided into three main categories: molecular function, biological process and cellular component. The identified functions and the corresponding numbers of DEGs for each GO category are shown

Gene ontology (GO) Slim terms contain specified sub-sets of higher-level ontology categories that provide a broad profile for genome-genome comparison [25]. Significant GO categories were designated as those with a *P* - value <0.01. Of 2053 DEGs between MZ-2 and MZ-3, 1203 DEGs were successfully annotated with 57 GO terms, 904 DEGs were categorized into biological processes, 884 DEGs into molecular functions and 847 DEGs into cellular components (Fig. 3). For the molecular functions, the dominant sub-categories of catalytic activity and binding accounted for 44.64% and 44.70%, respectively. Cellular component sub-categories with the largest numbers of annotated unigenes included cell part (22.11%) and cell (22.11%). Among the biological processes, the dominant sub-categories included metabolic processes (32.87%) and cellular processes (25.72%).

**Fig. 4:**
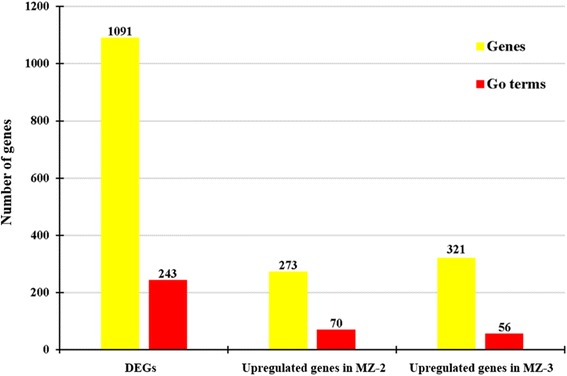
Gene Ontology (GO) enrichment of DEGs and upregulated genes. Bars show the number of DEGs and upregulated genes (*yellow bar*) and the number of annotated GO terms (*red bar*)

**Fig. 5:**
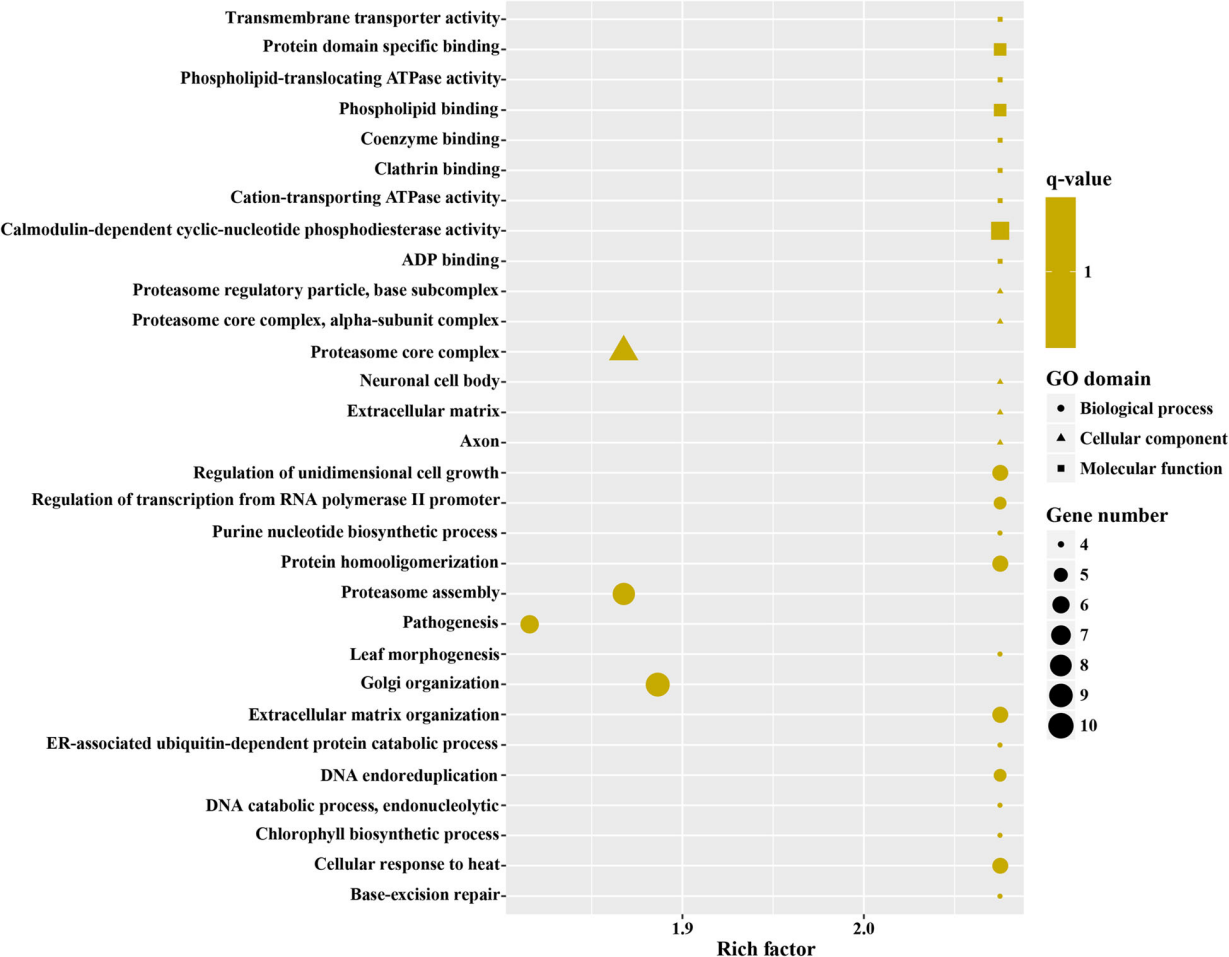
The top 30 of GO enrichment of significantly upregulated genes

GO is an international standardized gene functional classification system and was applied to search for significantly enriched GO terms in DEGs. To obtain a comprehensive view of DEGs, GO term enrichment analysis was carried out to evaluate significantly over-represented GO terms. Of 2053 DEGs between MZ-2 and MZ-3, 1091 DEGs were successfully annotated with 243 GO terms (Fig. 4), of which 509 DEGs were assigned to molecular function, 583 DEGs to biological process, and 522 DEGs to cellular component ontology. A large proportion of annotated DEGs involved in metabolic processes were novel genes related to pathways of secondary metabolite synthesis (Fig. 5).

**Fig. 6:**
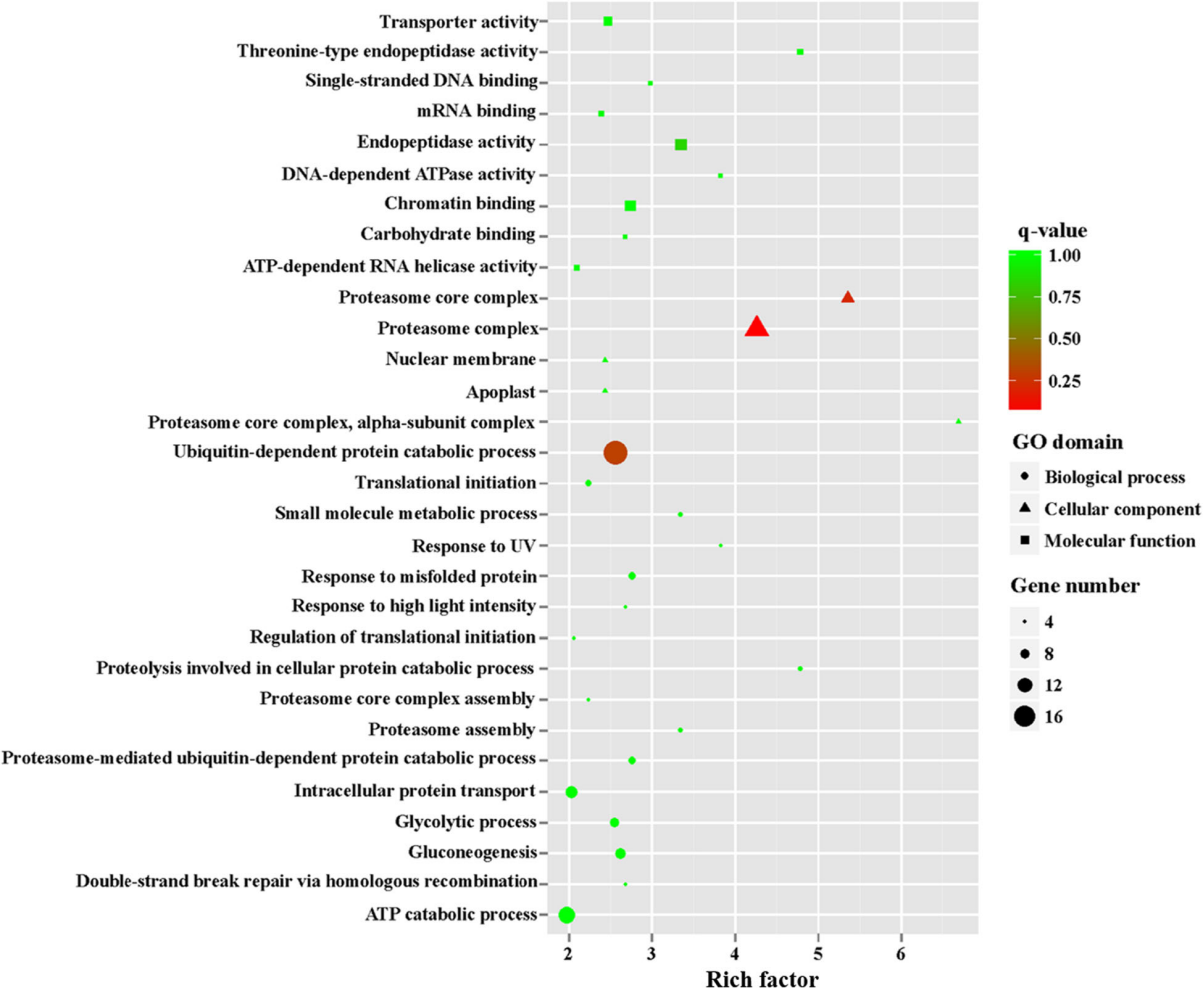
The top 30 of GO enrichment of significant upregulated genes in MZ-2

**Fig. 7:**
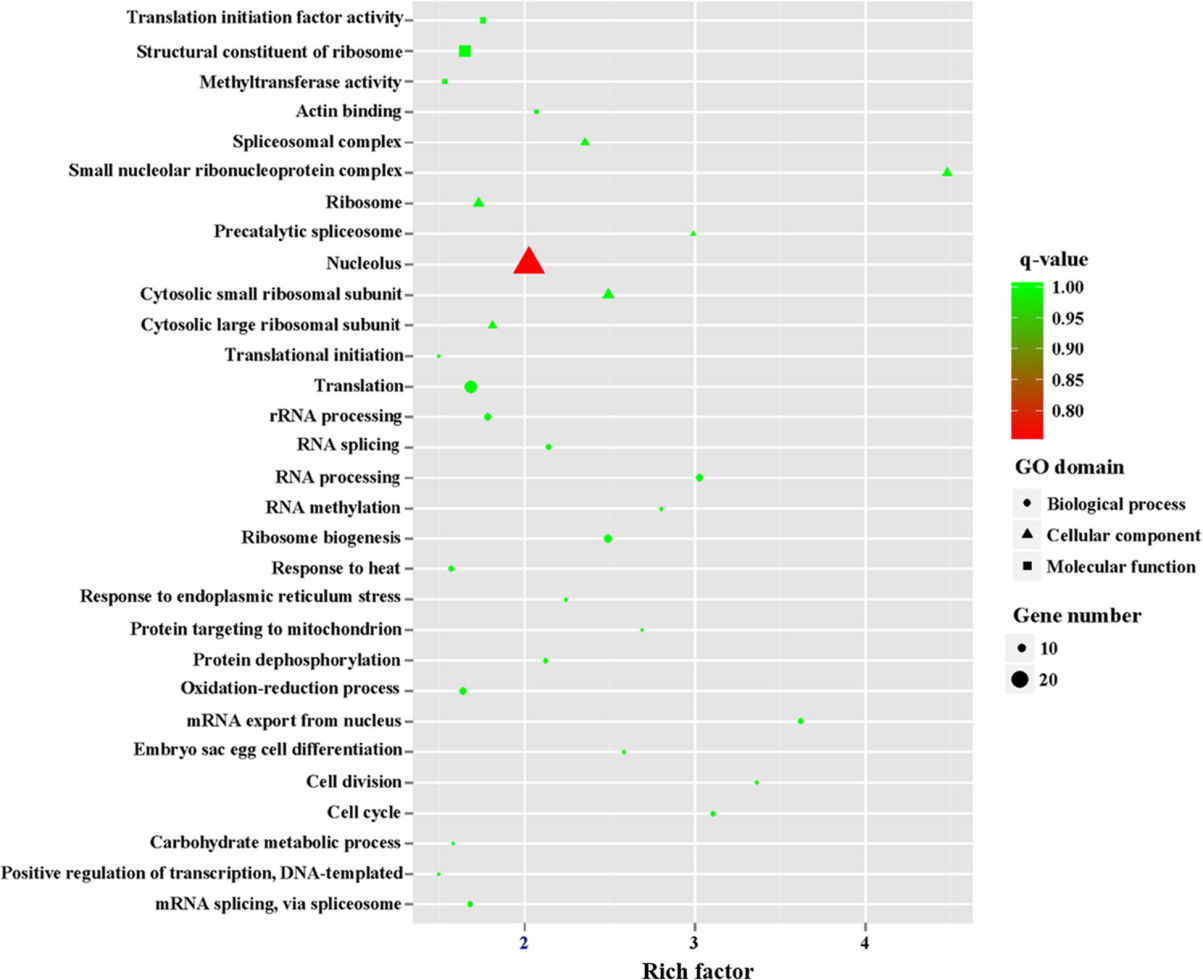
The top 30 of GO enrichment of significant upregulated genes in MZ-3

To obtain a detailed view of stage-specific upregulated genes, GO term enrichment analysis was carried out to evaluate significantly over-represented GO terms. Of 1216 upregulated genes in MZ-2273 upregulated genes in MZ-2 were categorized into 70 GO terms (Fig. 4), and the top 10 GO enrichments involved were in the proteasome core complex, alpha-subunit complex, proteasome core complex, proteasome complex, threonine-type endopeptidase activity, proteolysis involved in cellular protein catabolic process, DNA-dependent ATPase activity, response to UV, proteasome assembly, endopeptidase activity and small molecule metabolic processes (Fig. 6). Of 837 DEGs in MZ-3, 321 upregulated genes in MZ-3 were categorized into 56 GO terms (Fig. 4), and the top 10 GO enrichments involved were in the small nucleolar ribonucleoprotein complex, mRNA export from nucleus, nucleolus, cell division, cell cycle, RNA processing, precatalytic spliceosome, RNA methylation, protein targeting to mitochondria, embryo sac egg cell differentiation and ribosome biogenesis (Fig. 7).

### Biochemical pathways

**Fig. 8:**
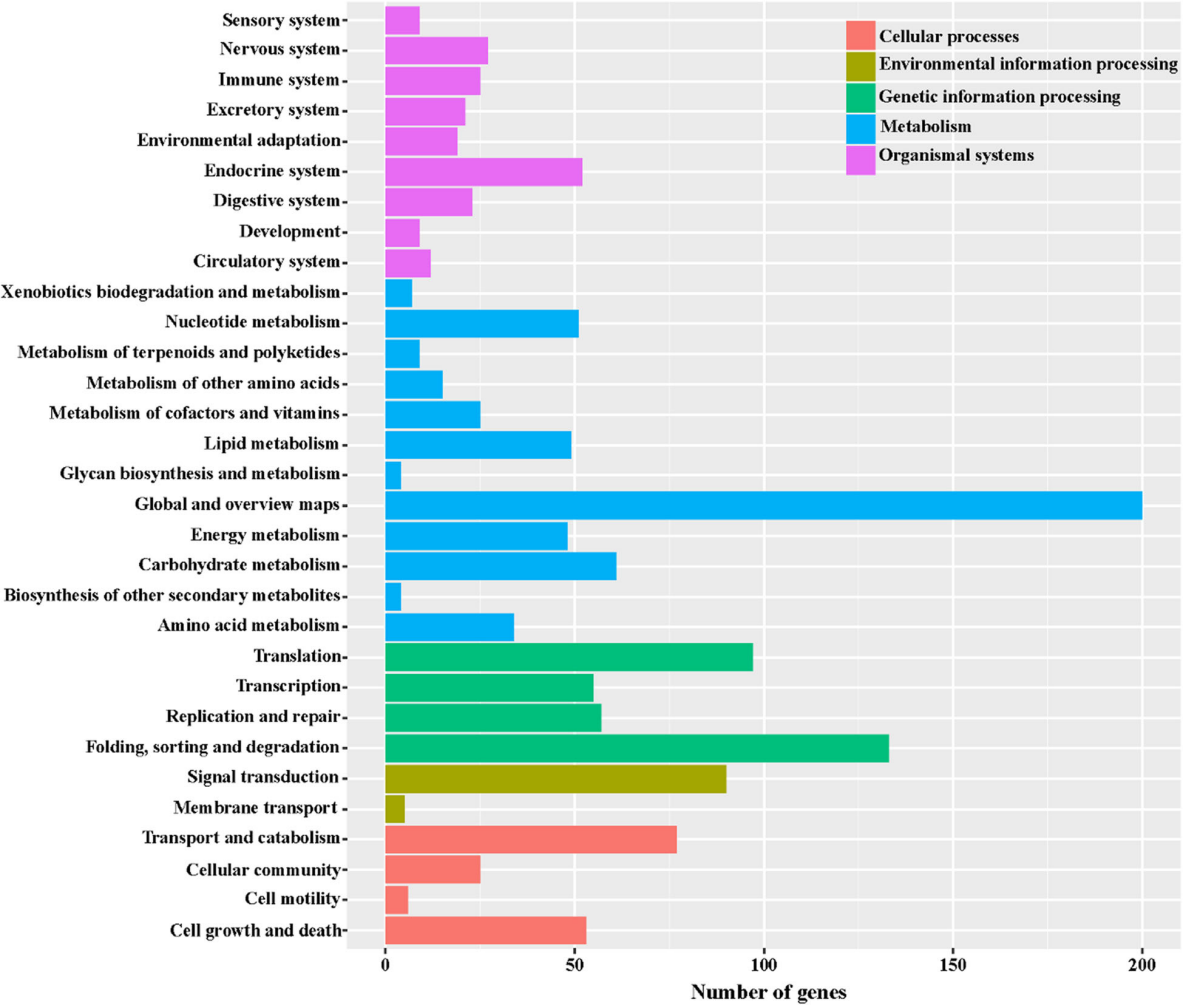
KEGG analysis of differentially expressed genes. The X-axis indicates the number of genes in the pathway. The Y-axis indicates the main pathways

KEGG analysis provides an alternative functional annotation based on genes associated with biochemical pathways. DEGs were mapped to the reference pathway in the KEGG database in order to identify the changes in biological pathways operating during the two developmental stages. The KEGG pathways were designated as those with a *P* - value < 0.05. Of the 2053 DEGs between MZ-2 and MZ-3, 620 unigenes were classified into 198 KEGG pathways (Fig. 8).

**Fig. 9:**
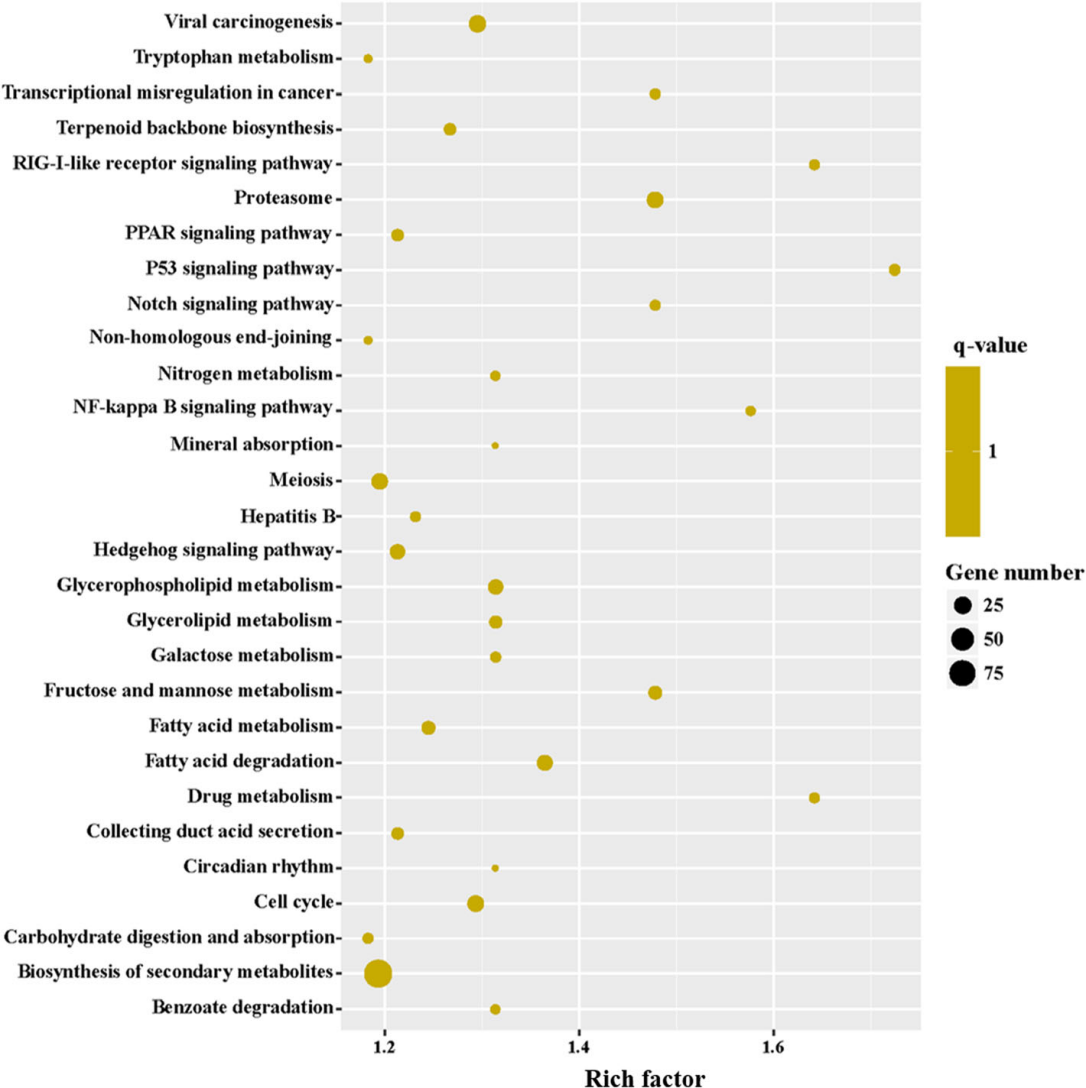
The top 30 of KEGG enrichment of significant upregulated genes

To obtain a detailed view of stage-specific upregulated genes, KEGG enrichment analysis was carried out to evaluate significantly over-represented KEGG terms. Of the 2053 DEGs between MZ-2 and MZ-3, 727 genes had a KO ID and were associated with 242 pathways. The KEGG metabolic pathways were well represented with the largest number of annotated DEGs (25.31%), followed by biosynthesis of secondary metabolites (12.65%) and biosynthesis of antibiotics (8.94%). The “p53 signaling pathway”, “drug metabolism”, “RIG-I-like receptor signaling pathway” and “NF-kappa B signaling pathway” were also highly enriched (Fig. 9).

**Fig. 10:**
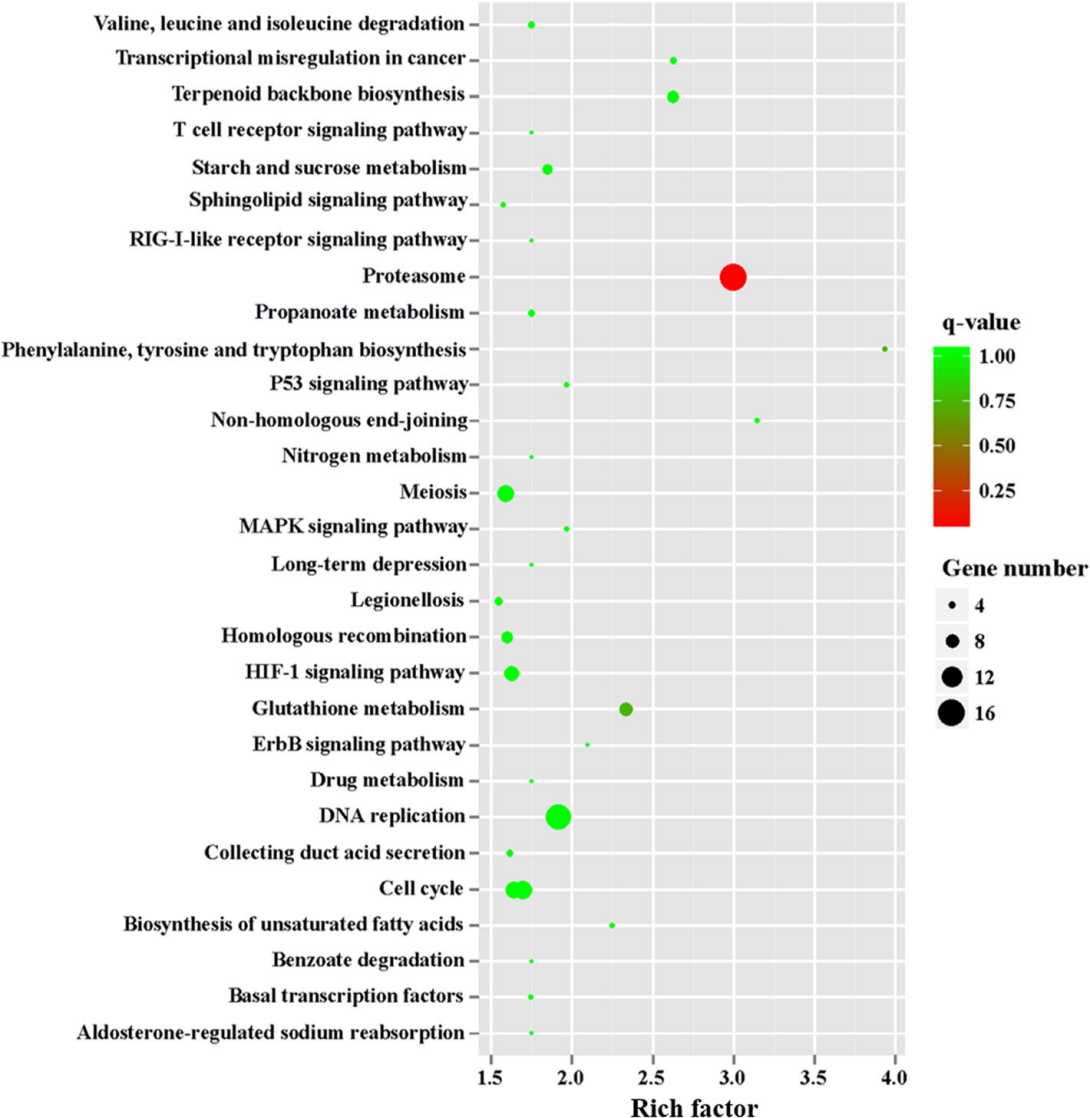
The top 30 of KEGG enrichment of significant upregulated genes in MZ-2

**Fig. 11:**
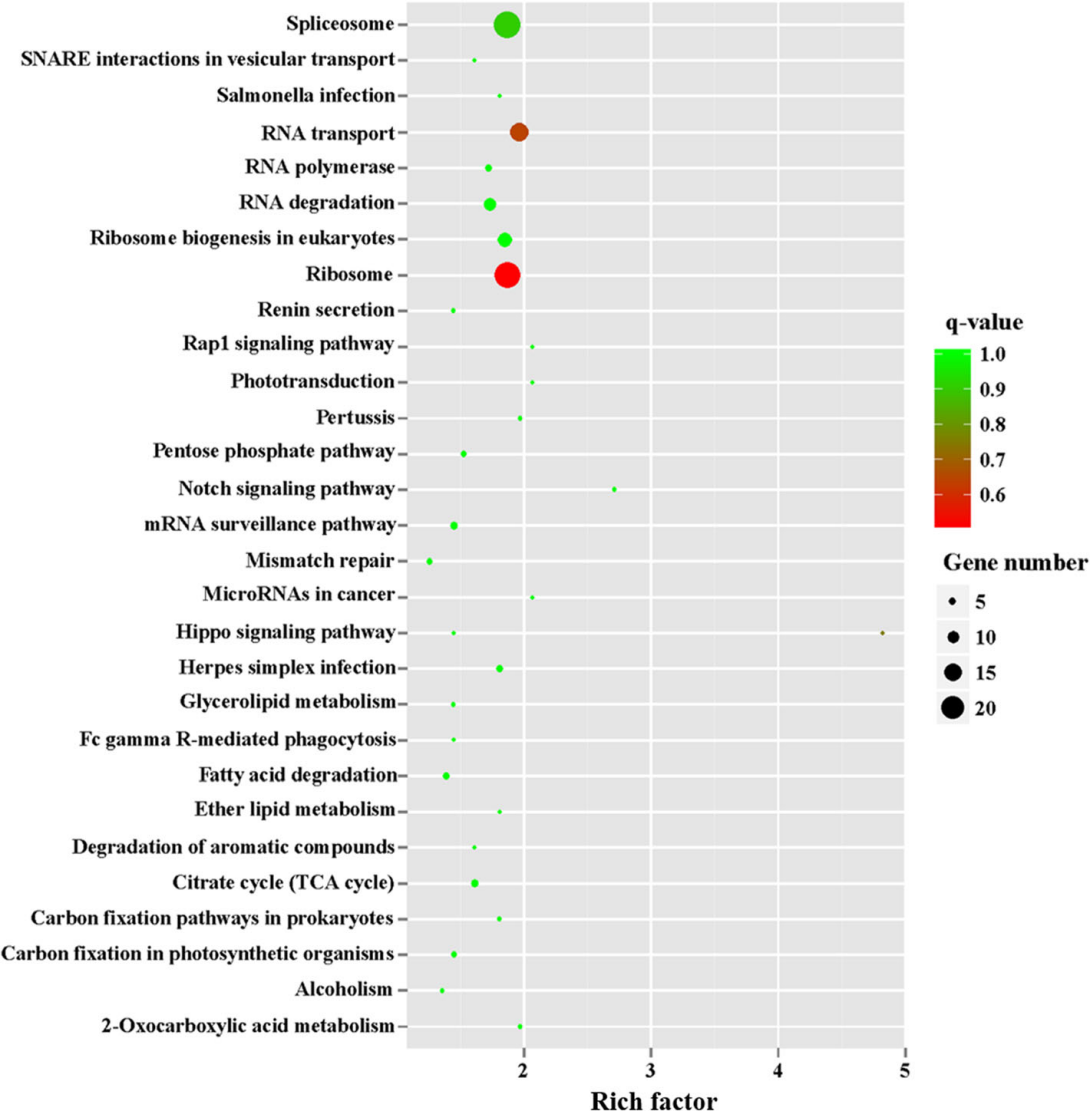
The top 30 of KEGG enrichment of significant upregulated genes in MZ-3

To further unveil changes of the pathways, we performed KEGG enrichment analysis of upregulated DEGs and downregulated DEGs. The 242 upregulated genes in MZ-2 were associated with 188 signal pathways, and the top 10 KEGG enrichments involved were for the proteasome, terpenoid backbone biosynthesis, DNA replication, phenylalanine, tyrosine and tryptophan biosynthesis, glutathione metabolism, non-homologous end-joining, transcriptional dysregulation in cancer, purine metabolism, cell cycle and biosynthesis of unsaturated fatty acids (Fig. 10). The 322 upregulated genes in MZ-3 were associated with 168 signal pathways, including RNA transport, ribosome, notch signaling pathway and the spliceosome. The top 10 KEGG enrichments involved were for the spliceosome, ribosome, RNA transport, hippo signaling pathway, ribosome biogenesis in eukaryotes, notch signaling pathway, RNA degradation, herpes simplex infection, 2-Oxocarbocylic acid metabolism and pertussis (Fig. 11).

### Quantitative real-time PCR validation of RNA-seq results

**Fig. 12:**
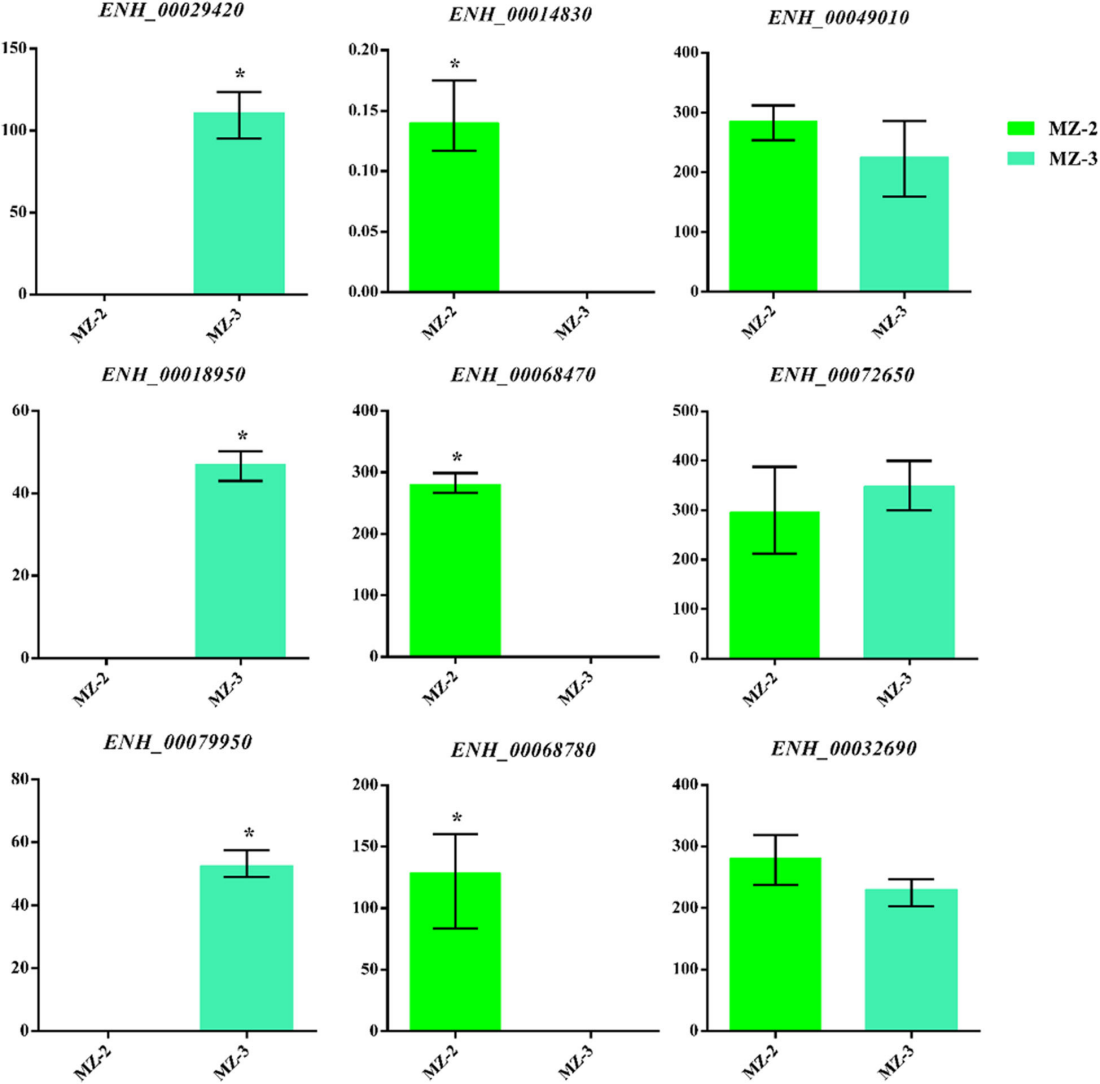
Validation of the RNA-Seq results by quantitative real-time PCR (qRT-PCR). Nine unigenes with significant differences and one unigene without significant difference were randomly selected for analysis using qRT-PCR. *GAPDH* was used for normalization. *Asterisks* represent significant difference (*P* < 0.05)

To validate the sequencing data, 9 differentially expressed mRNAs were further examined by qRT-PCR with gene-specific primer sequences. The expression levels were calculated according to the 2^-ΔΔCt^ values. According to the RNA-seq results, the expression levels of *ENH_00029420*, *ENH_00079950*, *ENH_00018950* were upregulated in MZ-3, the expression levels of *ENH_00068470*, *ENH_00068780*, *ENH_00014830* were downregulated in MZ-3, and the expression level of *ENH_00072650*, *ENH_00049010*, *ENH_00032690* were no significant difference. Using *GAPDH* as a reference gene, expression levels determined by qRT-PCR were consistent with those obtained by RNA-seq (Fig. 12), confirming the accuracy and reliability of the RNA-seq results. Thus, the data generated here can be used to investigate stage-specific expression of genes that show different expression levels among different developmental stages.

## Discussion

The whole genomes for all seven *Eimeria* species that infect chickens have been sequenced and annotated [26]. Genome-wide gene identification has predicted more than 8000 protein-coding genes in each of the hemorrhagic *Eimeria* genomes and *E. mitis*. For *E*. *necatrix*, the size of the genomic DNA is 55.2 Mb and encodes 8627 proteins [27]. In the present study, we investigated the transcript profiles of MZ-2 and MZ-3 of *E*. *necatrix* using RNA-seq and identified 6977 and 6901 genes in MZ-2 and MZ-3, respectively. The number of genes identified from *E*. *necatrix* was slightly more than that of *E. tenella*. By transcriptome sequencing, Reid et al. [27] identified expression of 76% of predicted *E. tenella* genes (6700) across four developmental life-stages (unsporulated oocyst, sporulated oocyst, sporozoite and merozoite).

The differential expression analysis of RNA-seq data in MZ-2 and MZ-3 revealed that 1216 genes were upregulated in MZ-2 and 837 genes were upregulated in MZ-3. Of 2053 DEGs, 95 and 48 genes were specifically expressed in MZ-2 and MZ-3, respectively. These genes included, for example, ApiAP2 DNA-binding proteins, *Eimeria*-specific surface antigens (SAGs), CMGC kinase, DEAD/DEAH box helicase domain-containing protein and proliferation-associated protein 2G4 (PA2G4) (Additional file 6: Table S5). The analysis of stage-specific expression genes revealed that 73 were hypothetical protein-coding genes of the 95 MZ-2-specific expression genes, and 35 were hypothetical protein-coding genes of the 48 MZ-3-specific expression genes. Since hypothetical proteins are not annotated in the database and not validated in other reports, we will not discuss them in this article. There are 8 structural protein-coding genes among the 15 MZ-3-specific expression genes that were mainly associated with cell morphology, mechanical support, defense, protection and repair. Compared to MZ-3, of the 22 MZ-2-specific expression genes, 15 were functional protein-coding genes, which were related to the biological functions such as metabolic, single-organism and cellular processes, and the catalytic activity, binding functions, and meanwhile, biosynthesis of secondary metabolites and metabolic pathways were also involved. These results revealed the functional differences from the specific expression genes between MZ-2 and MZ-3.

The apicomplexan AP2 (ApiAP2) family of DNA binding proteins was identified as a major class of transcriptional regulators that are found across all Apicomplexa that modulate key regulatory decisions at all stages of parasite development and stress-regulated gene expression in plants [28–30]. AP2 domain proteins play an important role in the transition from asexual to sexual replication in *Plasmodium* [31], *Cryptosporidium parvum* [32], *Toxoplasma gondii* [33] and *Theileria annulata* [34]. In *Eimeria*, the number of genes containing ApiAP2 domains was found to vary from 44 to 54, including 21 *Eimeria*-specific ApiAP2 groups, 22 additional groups shared by *Eimeria* and other coccidia, and five pan-apicomplexan clusters [27]. In our study, we detected 14 genes containing ApiAP2 domains, of which 12 were upregulated in MZ-2 and 2 were upregulated in MZ-3. This difference in expression profiles for ApiAP2 domain-encoding genes could be associated with the developmental fates of MZ-2 and MZ-3. Previous work on the merogony of *T. annulata* in an in vitro system found that the probability of merogony occurring could be increased by inhibition of DNA synthesis, while the inhibition of protein synthesis reduced the potential to reach commitment [35].

The principal surface antigen gene family in *E. tenella* is *sag*, which encodes single domain, membrane-bound proteins tethered by glycosylphosphatidylinositol (GPI) anchors to the surface of invasive sporozoites and merozoites [36]. The total number of *sag* genes varies greatly among the seven avian *Eimeria* species, from 19 *sags* (*E. praecox*) to 172 *sags* (*E. mitis*). *Eimeria tenella* has 89 *sags* that are further divided into three subfamilies, including *sagA*, *sagB* and *sagC*. The expression profiles for the *sagA* and *sagB* subfamilies varied in different developmental stages of *E. tenella*; a small number of *sagA* genes peaked in expression at each stage, while *sagB* genes all peaked in expression in second-generation merozoites [27]. There are 119 *sag* genes and 102 pseudogene fragments in the *E. necatrix* genome [27]. In this study, a total of 99 *sags* (88 *sag*s in MZ-2, 98 *sag*s in MZ-3) were detected. Of 87 *sags* expressed in both MZ-2 and MZ-3, 64 *sags* displayed upregulation that only occurred in MZ-3. Of 64 *sags*, two *sags* were specifically expressed in MZ-3. This difference in expression profiles for *sag* genes could be associated with the developmental fates of MZ-2 and MZ-3. In the life-cycle of *E. necatrix*, MZ-2 liberated from the second-generation meronts pass to the cecum, where they penetrate the epithelial cells and develop into third-generation meronts. Thus, the reduced expression of some *sags* in MZ-2 may be beneficial for parasites to escape from host immune responses. In contrast to MZ-2, MZ-3 liberated from the third-generation meronts immediately enters into other cecal epithelial cells near the cecal tonsil, which contains numerous lymphocytes, and become macrogametes or microgamonts. The increased expression of some *sags* in MZ-3 may help parasites to attach to the host cells prior to parasite invasion.

CMGC proteins participate in several signaling pathways critical for development processes and cell homeostasis, and DEAD/DEAH box proteins play crucial roles in almost all aspects of RNA metabolism, including transcription, splicing, translation and decay [37–39]. It has been proposed that CMGC kinase has evolved independently within the Apicomplexa to provide specialized functions related to life-cycle transitions [40]. Compared to MZ-3, 4 genes coding for CMGC kinase and 4 genes coding for DEAD/DEAH box helicase domain-containing protein were detected in MZ-2.

Proliferation-associated protein 2G4 (PA2G4), also known as ErbB3-binding protein 1 (EBP1), is implicated in cell growth, differentiation and apoptosis. Recent reports have shown that EBP1 participates in the regulation of intestinal inflammation *via* mediating the Akt signaling pathway, and the downregulation of EBP1 could accelerate intestinal inflammation [41]. In this study, we found that EBP1 was upregulated in MZ-2. The upregulation of EBP1 in MZ-2 suggested that parasites could escape immune responses by attenuating inflammatory damage.

GO enrichment analysis showed that the molecular functions of upregulated genes in MZ-2 and MZ-3 were different. The molecular functions of upregulated genes in MZ-2 were mainly enriched in proteasomal degradation, which is essential for many cellular processes, including the cell cycle, the regulation of gene expression, and responses to oxidative stress. The proteasome plays a straightforward but critical role in the function of the adaptive immune system. The molecular functions of upregulated genes in MZ-3 were mainly enriched in transcription, cell division, cell differentiation and RNA splicing *via* the spliceosome. The KEGG enrichment analysis also revealed that the molecular functions of upregulated genes in MZ-2 and MZ-3 were different. The molecular functions of upregulated genes in MZ-2 were mainly enriched for protein degradation and amino acid metabolism. The molecular functions of upregulated genes in MZ-3 were mainly enriched for transcriptional activity, cell proliferation and cell differentiation.

## Conclusions

The present study represents the first report on a large-scale analysis of differentially expressed genes between MZ-2 and MZ-3 of *E*. *necatrix* using RNA-seq. Our study adds a number of genes to the list of candidate genes involved in the reproductive mode plasticity of *Eimeria*. Thus, these results provide a comprehensive gene expression profile among asexual reproduction stages of *E*. *necatrix*, and will extend our knowledge of the putative regulation of sex differentiation and development in *Eimeria*. Further studies on the candidate genes will help to unfold the molecular mechanisms underlying the transition from MZ-2 to MZ-3 of the *E*. *necatrix* as well as to develop novel strategies for coccidiosis control.

## Additional files

Additional file 1: Figure S1. The purified MZ-2 and MZ-3. **a** A MZ-2 sample purified from *E*. *necatrix*-infected chickens at 136 h post-infection. **b** A MZ-3 sample purified from *E*. *necatrix*-infected chickens at 144 h post-infection. *Scale-bars*: 10 μm. (TIFF 829 kb)

Additional file 2: Table S1. Primers used in qRT-PCR. (XLSX 11 kb)

Additional file 3: Table S2. Information on DEGs in the pair-wise comparison of MZ-2 and MZ-3. (XLSX 209 kb)

Additional file 4: Table S3. Information on MZ-2-specific mRNA transcripts. (XLSX 18 kb)

Additional file 5: Table S4. Information on MZ-3-specific mRNA transcripts. (XLSX 15 kb)

Additional file 6: Table S5. Information on differentially expressed mRNA transcripts coding for SAG proteins, CMGC, ApiAP2 and DEAD BOX. (XLSX 36 kb)

## Abbreviations

ApiAP2: Apicomplexan AP2
DE: Differential expression
DEG: Differentially expressed gene
FPKM: Fragment per kilobase of exon model per million mapped reads
GO: Gene ontology
GPI: Glycosylphosphatidylinositol anchor
KEGG: Kyoto Encyclopedia of Genes and Genomes
MZ-2: Second-generation merozoites
MZ-3: Third-generation merozoites
PA2G4: Proliferation-associated protein 2G4
qRT-PCR: Relative quantitative real-time PCR
*sag*: Surface antigen gene
SRS: SAG1 related sequences
