# Supplementary figures and images for: Comparative transcriptome analysis of second- and third-generation merozoites of Eimeria necatrix
Source: Parasit Vectors. 2017 Aug 16;10:388. doi: 10.1186/s13071-017-2325-z (PMC5559809; doi:10.1186/s13071-017-2325-z)

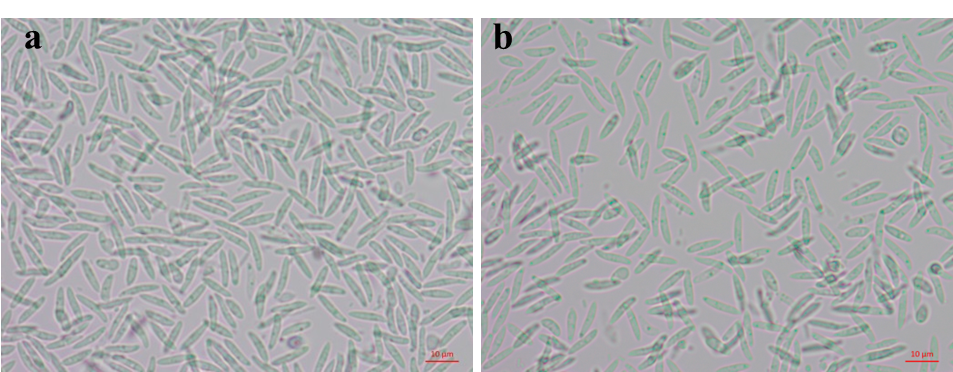

Supplement: Supplementary file 1 — The purified MZ-2 and MZ-3. a A MZ-2 sample purified from E. necatrix-infected chickens at 136 h post-infection. b A MZ-3 sample purified from E. necatrix-infected chickens at 144 h post-infection. Scale-bars: 10 μm. (TIFF 829 kb) [file 13071_2017_2325_MOESM1_ESM.tif]
